# Theoretical Study of Ultra-Fast Laser Lift-Off of Carbon Nanotube-Integrated Polyimide Films

**DOI:** 10.3390/nano16010001

**Published:** 2025-12-19

**Authors:** Run Bai, Yachong Xu, Junwei Fu, Zhenzhen Sun, Yanbo Wang, Rui Yang, Zijuan Han, Fanfan Wang, Boyuan Cai

**Affiliations:** 1 School of Artificial Intelligence Science and Technology, University of Shanghai for Science and Technology, Shanghai 200093, China; 232200331@st.usst.edu.cn (R.B.); 243350583@st.usst.edu.cn (Y.X.); 233350708@st.usst.edu.cn (J.F.); 243350694@st.usst.edu.cn (Z.S.); 241200265@st.usst.edu.cn (Y.W.); 2 Institute of Photonic Chips, University of Shanghai for Science and Technology, Shanghai 200093, China; 3 Shenzhen Han’s Semiconductor Equipment Technology Co., Ltd., Shenzhen 518101, China; yangr120184@hanslaser.com (R.Y.); wangff@hanslaser.com (F.W.); 4 School of Mechatronic Engineering, Guangdong University of Technology, Guangzhou 510006, China; hanzijuan@mails.gdut.edu.cn

**Keywords:** ultra-fast laser lift-off, two-temperature model, carbon nanotubes, polyimide films

## Abstract

In this paper, ultra-fast laser lift-off (LLO) of carbon nanotube (CNT)-integrated polyimide film (PI) was investigated by different laser burst mode and pulse intervals using the two-temperature model. By comparing the temperature field distributions of nanosecond, picosecond, and femtosecond lasers at different pulse intervals, it can be found that picosecond lasers cause a higher lattice temperature increase at the PI interface with specific pulse interval conditions. With the increase in the pulse interval, the lattice temperature of the three kinds of lasers decreased, indicating that the heat accumulation effect was weakened. In addition, under picosecond laser irradiation, the lattice temperature at the PI/glass interface of integrated CNTs could be significantly increased, which was significantly different from the system without integrated CNTs. The simulation results show that the picosecond laser is more suitable for LLO with an appropriate pulse interval, and the integration of CNTs at the PI/glass interface can effectively reduce the laser energy threshold required for the LLO process. Our work presents a new PI/CNT/glass model for ultra-fast laser low-threshold LLO and promotes the laser debonding technology in the fields of OLED and other optoelectronic chips.

## 1. Introduction

In recent years, with the rapid development of information technology, flexible electronic devices have shown great potential in the fields of biomedical diagnosis, wearable devices, and flexible display due to their unique advantages, such as flexibility, extensibility, and curved surface integration [1,2]. However, the mechanical vulnerability of flexible substrates makes it difficult to achieve direct compatibility with high-precision micro-fabrication processes [3,4]. Traditional lift-off technologies, such as mechanical lift-off technology [5,6] and the chemical etching method [7,8], struggle to meet the compatibility requirements due to the limitations of stress concentration and the complexity of the process. Therefore, laser lift-off (LLO) [9,10] has become an effective solution to the above problems. LLO utilizes the thermoelastic effect of pulsed laser to directionally irradiate the sacrificial layer at the interface through the transparent substrate to induce its thermal decomposition or phase transition, leading to the separation of flexible devices [11,12]. At present, LLO is being used to peel a variety of solid materials, including gallium nitride (GaN) [13,14], lead zirconate titanate (PZT) [15,16], amorphous silicon (a-Si) [17], etc. In addition, the technology has also been widely used in the field of flexible device lift-off, such as in relation to polyimide (PI), which is a key material for the fabrication of commercial flexible electronic devices [18,19].

However, traditional LLO uses a laser with high energy density to ablate the sacrificial layer, which is prone to heat shock, leading to the structural fracture of ultra-thin flexible devices arising from thermal damage [20]. In addition, traditional LLO cannot accurately control the interface adhesion [21], nor can it control the surface morphology after debonding [22,23]. These limitations limit the application of LLO in the field of flexible electronic devices. In order to solve these problems, many innovative methods have been proposed, such as multiple scanning LLO technology (m-LLO) [24] and laser-induced interface lift-off technology (LIIS) [25]. Although these technologies have improved the debonding efficiency to some extent, the thermal mechanism of ordinary lasers still causes thermal damage during the LLO process [26]. To solve this problem, researchers proposed using ultra-fast laser to reduce thermal damage in the LLO process. The pulse duration of ultra-fast laser is very short, usually less than $10^{-9}$ s. When interacting with materials, because the pulse width is far lower than the thermal diffusion time of the materials, the laser energy can be absorbed and localized in a very short time [27]. Meanwhile, the interaction between ultra-fast laser and materials can directly destroy chemical bonds or lattice structures through nonlinear absorption effects [28]. These characteristics enable ultra-fast laser to achieve cold processing with almost no heat effects, which may further reduce thermal damage in the LLO process [29,30]. Currently, a variety of theoretical models have been established and widely studied for the LLO process of solid materials by ultra-fast laser [31]. However, there is still a lack of systematic research on the light/material interaction effects of the different ultra-fast lasers with the PI film and how to reduce the ultra-laser energy threshold by integrating nanomaterials.

In this study, we established a PI/CNT/glass [32,33] LLO model including dynamic optical and thermophysical properties based on two-temperature equations to study the non-equilibrium energy transfer of the PI film irradiated by different types of ultra-fast lasers (ns, ps, and fs lasers). The influence of the pulse interval of the different ultra-fast lasers on the electron and lattice temperature of the PI film during the LLO process was investigated. The simulation results show that for the PI/CNT/glass model the picosecond laser is more suitable for the debonding process of the PI interface compared with the nanosecond and femtosecond lasers, and the heat accumulation of two adjacent sub-pulses decreases with the increase in the sub-pulse separation time, which can slow down the rate of the lattice temperature increase. In addition, the integration of carbon nanotubes at the PI/glass interface can effectively enhance the interface temperature, thereby reducing the laser threshold required for the LLO process and the thermal damage. From the comparison, the increase in the lattice temperature of the PI film with CNTs integrated is around 2000 K higher than that without CNTs. Our findings may offer a new model for utilizing ultra-fast lasers and nanomaterials for a low-threshold LLO process.

## 2. Numerical Model

### 2.1. Two-Temperature Model

In most previous cases, the lattice heat transfer is usually ignored because only one single pulse is considered in the simulation. However, in practical situations, many sub-pulses are commonly applied during the LLO process. To guide the real processing of the related materials, especially for real industrial processing, here the two-temperature model (TTM) is utilized for investigating the influence of the laser burst mode with sub-pulses in the LLO process. The TTM is a theoretical model used to describe the non-equilibrium thermal dynamics of electrons and lattices in the process of interaction between ultra-fast laser and materials. With an ultra-fast pulse laser, the energy absorbed by the material first heats the electron rapidly, while the lattice cannot respond immediately due to thermal inertia, resulting in a significant temperature difference between the electron and the lattice and forming an electron lattice non-thermal equilibrium state. Equation [34] of the TTM is shown as Equations (1) and (2):

(1) $$C_{e}\frac{\partial T_{e}}{\partial t}\;=\;\frac{\partial }{\partial x}\left(k_{e}\frac{\partial T_{e}}{\partial x}\right)\;+\;\frac{\partial }{\partial z}\left(k_{e}\frac{\partial T_{e}}{\partial z}\right)-G(T_{e}-T_{l})+I(x,z,t)$$

(2) $$C_{l}\frac{\partial T_{l}}{\partial t}\;=\;\frac{\partial }{\partial x}\left(k_{l}\frac{\partial T_{l}}{\partial x}\right)\;+\;\frac{\partial }{\partial z}\left(k_{l}\frac{\partial T_{l}}{\partial z}\right)+G(T_{e}-T_{l})$$

(3) $$C_{e}=\frac{3k_{B}n_{e}}{2}$$

(4) $$k_{e}=\frac{2k_{B}^{2}n_{e}\mu_{e}T_{e}}{e}$$

(5) $$C_{l}=3k_{B}n_{0}$$

where $C_{e}$ is the heat capacity of the free electron, $C_{l}$ is the heat capacity of the lattice, $T_{e}$ is the electron temperature, $T_{l}$ is the lattice temperature, $k_{e}$ is the electronic conductivity, $k_{l}$ is the lattice thermal conductivity, $G=C_{e}/\tau_{e}$ is the electron–lattice coupling factor with $\tau_{e}$ of the characteristic electron–lattice energy, *t* is time, *x* is the coordinates of position relative to the center of the laser spot, *z* is the depth from the surface of the bulk material, $k_{B}$ is the Boltzmann constant, $n_{e}$ is the free electron density, $\mu_{e}$ is the electron mobility, *e* is the electronic charge, and $n_{0}$ is the atomic number density. $I\left(x,z,t\right)$ is the sectional energy term, which can be expressed by the following formula:

(6) $$I(x,z,t)=I_{0}(t)\alpha (x,z,t)[1-R(x,z,t)]\times \exp\left[-\frac{2{\left(x-x_{0}\right)}^{2}}{r_{0}^{2}}-\alpha (x,z,t)z\right]$$

where $I_{0}\left(t\right)$ is the time distribution of the laser beam, which can be expressed as

(7) $$I_{0}(t)=\frac{2}{\sqrt{\frac{\pi }{In2}}}\times \frac{F}{n\cdot t_{p}}\times \sum_{i=1}^{n}\exp\left[-(4In2){\left(\frac{t-2t_{p}-(i-1)\Delta t}{t_{p}}\right)}^{2}\right]$$

where $R\left(x,z,t\right)$ is reflectivity, $\alpha \left(x,z,t\right)$ is the absorption coefficient, ∆t is the sub-pulse separation time, *n* is the number of sub-pulses, *F* is the total laser fluence in a burst, $x_{0}$ is the coordinates of the laser spot center, and $t_{p}$ is the pulse duration defined by the full width at half maximum.

### 2.2. Simulation Model

**Figure 1:**
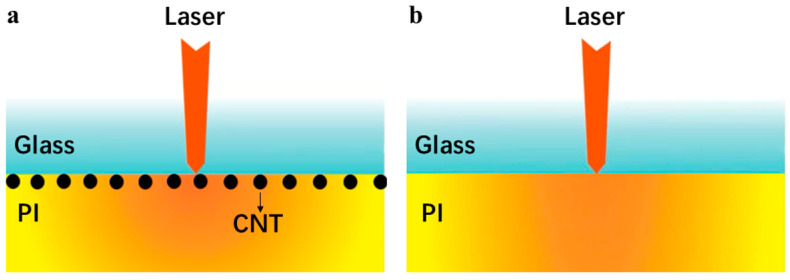
PI/glass interface model (**a**) with CNTs integrated; (**b**) without CNTs integrated.

In order to better study the effect of the CNTs on the lattice temperature of the PI interface with ultra-fast laser irradiation, we established a simulation model using COMSOL Multiphysics (version 6.3 of COMSOL Multiphysics) with CNTs integrated between the PI and the glass interface. As shown in Figure 1a, a carbon nanotube (CNT) layer was inserted at the PI/glass interface to improve the local heat absorption as a high absorption coefficient material. The TTM was utilized to couple dynamic optical and thermophysical parameters to describe the electron phonon non-equilibrium energy transfer process. The high thermal diffusion ability of CNT can significantly improve the interface temperature, similar to the CNT “heating wire” effect. A model without CNTs was also built for comparison, as shown in Figure 1b. The thickness of the PI film was set to 500 nm and the diameter of the CNT was 200 nm with a period of 400 nm. PI has a relatively low thermal conductivity (approximately 0.1–0.3 W/(m·K). In our model, the thermal conductivity of PI is set to 0.2 W/(m·K), the density is 1.42 g/cm^3^ with an absorption coefficient of 2 × 10^5^ cm^−1^, and the specific heat capacity is 800 J/(kg·K). CNTs have an extremely high thermal conductivity along the axial direction (>3000 W/(m·K)). In our model, the thermal conductivity of CNTs is 3500 W/(m·K), the density of CNTs is 1 g/cm^3^, and the specific heat capacity is 700 J/(kg·K). Glass also has a relatively low thermal conductivity (approximately 0.8–1.2 W/(m·K)). In our model, the thermal conductivity of glass is taken as 1 W/(m·K), the density is set at 2.37 g/cm^3^, and the specific heat capacity is 700 J/(kg·K) [35,36].

## 3. Results and Discussion

When an ultra-fast laser pulse irradiates on the PI material, the laser energy is first absorbed by free electrons, and the electrons reach a quasi-equilibrium state through the collision between electrons in a very short time, forming an extremely high-temperature electron gas. Then, the high-temperature electron gas transfers the energy to the lattice through collision with phonons, causing the lattice temperature to rise. The lattice temperature is the direct physical cause of phase transition, ablation, or bonding failure of the material. When the lattice temperature of the material reaches the decomposition threshold of the bonding layer, the chemical bond can break to realize debonding and finally realize the LLO of the material.

The temperature evolution irradiated by a single burst consisting of three sub-pulses was studied with different types of ultra-fast lasers: nanosecond laser, picosecond laser, and femtosecond laser. The effects of the laser pulse intervals on the electron and lattice temperature of the PI film with CNTs were also investigated. Since we only investigated the effects of three different ultra-fast lasers and different pulse intervals on the electron and lattice temperature at the PI interface, the pulse widths of the three types of lasers in the LLO process were selected as 20 ns, 20 ps, and 100 fs, according to the practical experimental conditions. The wavelengths of the three different ultra-fast lasers are 532 nm, 355 nm, and 780 nm respectively, with consistent laser energy (1 J/cm^2^). Our model employs ultra-fast laser irradiation of the PI/CNT/glass interface, generating transient high temperatures with three pulses, exceeding the melting point of PI (approximately 700–800 K). However, this transient high temperature is insufficient to induce melting of the large-area macroscopic material; instead, it induces thermal decomposition of PI at the interface (the decomposition temperature of PI is approximately 1200 K), ultimately leading to PI debonding.

**Figure 2:**
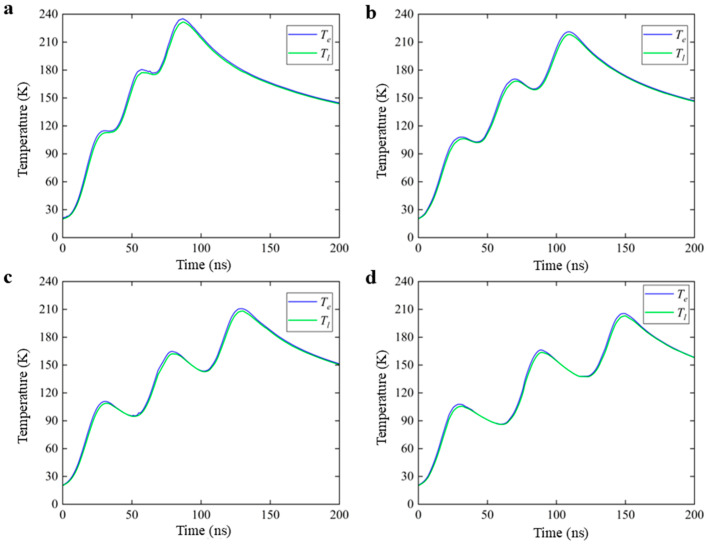
Surface temperature of electron and lattice under 3-sub-pulse nanosecond laser irradiation with pulse intervals of (**a**) 30 ns, (**b**) 40 ns, (**c**) 50 ns, and (**d**) 60 ns. In the figure, $T_{e}$ represents the electron temperature and $T_{l}$ represents the lattice temperature.

Figure 2 shows the variations in the electron and lattice temperature at the PI/glass interface when irradiated by the nanosecond laser with a pulse width of 20 ns and pulse intervals of 30 ns, 40 ns, 50 ns, and 60 ns. As seen in Figure 2a, the lattice temperatures with three pulses increased by 110 K, 180 K, and 230 K, and there is no obvious decreasing trend in the lattice temperature. However, the lattice temperature after three consecutive pulses begins to show a downward trend with the pulse interval increasing, as illustrated in Figure 2b–d. Meanwhile, the lattice temperature starts to drop significantly between the two consecutive sub-pulses and it drops rapidly at the end of each pulse. The lattice temperature can only increase by about 200 K at most when the pulse interval is increased to 60 ns. It can be found that increasing the pulse interval is not conducive to the heat accumulation between ns laser pulses, and the maximum lattice temperature gradually decreases with the increase in the sub-pulse separation time.

**Figure 3:**
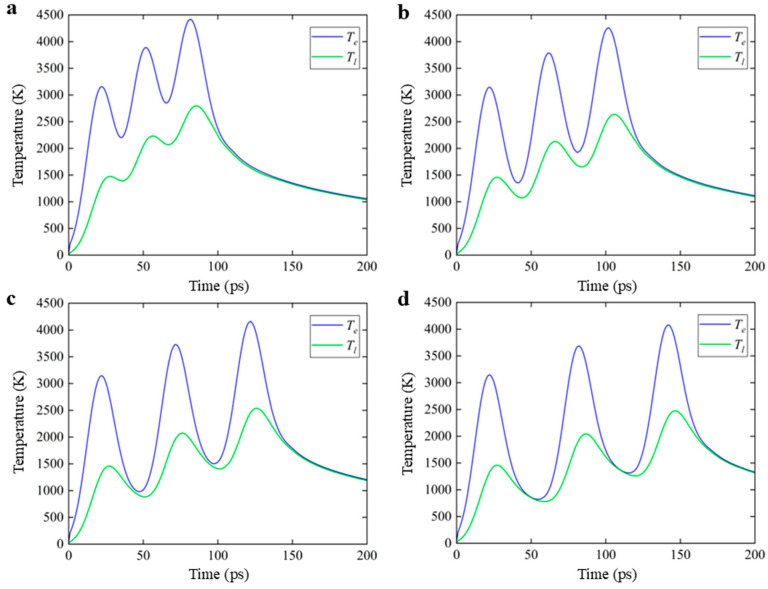
Surface temperature of the electron and lattice under 3-sub-pulse picosecond laser irradiation with pulse intervals of (**a**) 30 ps, (**b**) 40 ps, (**c**) 50 ps, and (**d**) 60 ps. In the figure, $T_{e}$ represents the electron temperature and $T_{l}$ represents the lattice temperature.

Figure 3 shows the distribution of the electron and lattice temperature at the PI/glass interface irradiated by the picosecond laser with a pulse width of 20 ps, and the pulse intervals are set to 30 ps, 40 ps, 50 ps, and 60 ps. Compared with the case with nanosecond laser irradiation, the electron and lattice temperature under picosecond laser irradiation increased significantly. Under the same laser energy, from Figure 3a it can be found that the lattice temperatures with the three ps sub-pulses can be increased by about 1500 K, 2200 K, and 2800 K, which is much higher than the increment of the lattice temperature with nanosecond laser irradiation. With the pulse interval increasing, the maximum lattice temperature and the lattice temperature at the end of each pulse gradually decrease. The lattice temperature can only be increased by about 2500 K at most when the pulse interval increases to 60 ps. It can still be found that the heat accumulation effect of two adjacent pulses weakens as the pulse interval increases with picosecond laser irradiation.

**Figure 4:**
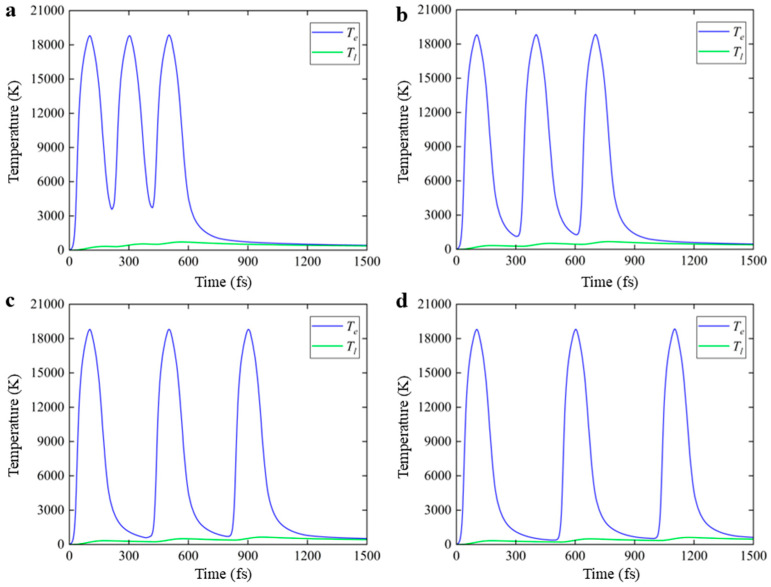
Surface temperature of electron and lattice under 3-sub-pulse femtosecond laser irradiation with pulse intervals of (**a**) 200 fs, (**b**) 300 fs, (**c**) 400 fs, and (**d**) 500 fs. In the figure, $T_{e}$ represents the electron temperature and $T_{l}$ represents the lattice temperature.

Figure 4 shows the variations in the electron and lattice temperature when the PI/glass interface is irradiated by the femtosecond laser with a pulse width of 100 fs and pulse intervals are set to 200 fs, 300 fs, 400 fs, and 500 fs. Different from the temperature change with the nanosecond and picosecond lasers’ irradiation, the electron temperature with femtosecond laser irradiation rises significantly after absorbing photon energy. The electron temperature can increase to nearly 19,000 K while the rise of lattice temperature lags significantly. The increase in the lattice temperature is only around 700 K, which is much lower than the debonding temperature threshold of the PI material. Therefore, femtosecond laser is not suitable for the PI film LLO. In addition, it can be found that the increase in the fs laser pulse intervals does not significantly influence the overall temperature rise tendency for both the electron and the lattice.

**Figure 5:**
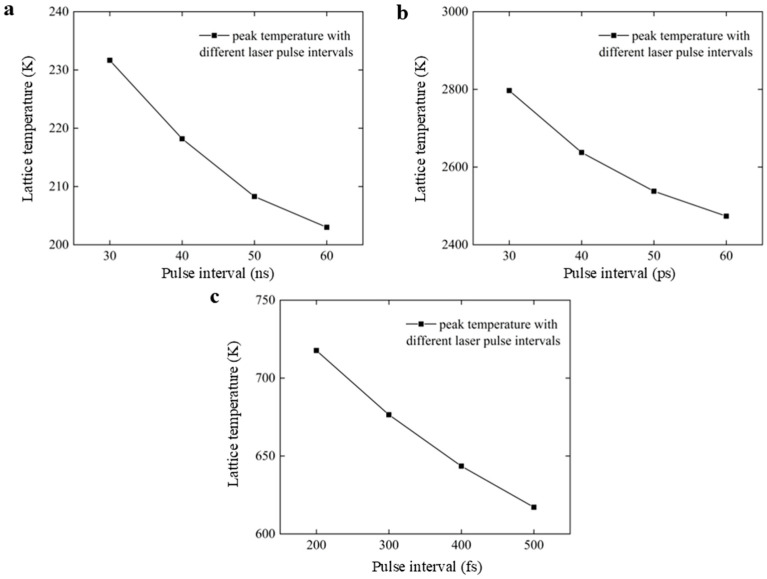
Peak temperature variation curves with different laser pulse intervals under (**a**) ns, (**b**) ps, and (**c**) fs lasers.

In order to further illustrate the variation trend of peak lattice temperature with pulse interval for three different ultra-fast lasers with three-pulse irradiation, we plotted the peak temperature variation curves of ns, ps, and fs lasers with different laser pulse intervals based on the peak lattice temperature data. As shown in Figure 5, the peak lattice temperature of the three ultra-fast lasers with three-pulse irradiation all decreased with the increase in the pulse interval. It can be found that for three different types of ultra-fast lasers, increasing the pulse interval will reduce the thermal accumulation effect between pulses, ultimately leading to a decrease in the peak lattice temperature.

Comparing the lattice temperature variations irradiated by three different ultra-fast lasers (ns, ps, and fs lasers) with different pulse intervals at the PI/glass interface, it can be found that with nanosecond laser irradiation, the energy loss in the heat transfer process between electrons and the lattice is extremely low but the maximum lattice temperature cannot reach the debonding temperature of the PI film. Under femtosecond laser irradiation, the lattice fails to obtain sufficient energy due to the short pulse duration, resulting in a very slow rise in lattice temperature. Therefore, neither of them is suitable for the PI/CNT/glass model LLO process. However, the situation with picosecond laser irradiation is significantly different. The energy loss during the heat transfer process between the electron and the lattice with picosecond laser irradiation is relatively low. Meanwhile, the laser energy can be efficiently stored in the lattice system due to the matching of picosecond pulse width and electron/lattice energy relaxation time, causing a significant increase in lattice temperature and successfully exceeding the debonding threshold of the PI material.

**Figure 6:**
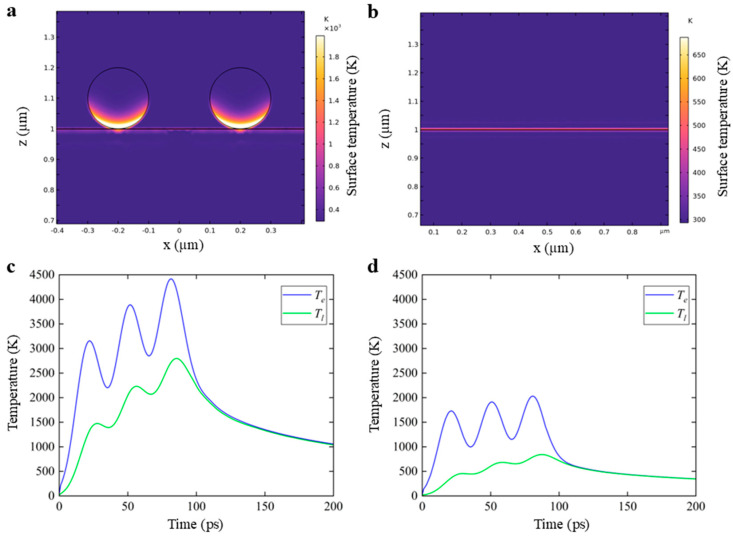
The temperature distributions of the model with CNTs integrated at the PI/glass interface (**a**,**c**); without CNTs integrated (**b**,**d**). In the figure, $T_{e}$ represents the electron temperature and $T_{l}$ represents the lattice temperature.

In order to further investigate the effect of CNTs on ultra-fast laser lift-off of the PI film, the comparison of the temperature distribution of the PI surface with/without CNTs integrated was performed, as illustrated in Figure 6. The model was irradiated by the picosecond laser with a pulse width of 20 ps and a pulse interval of 30 ps.

As shown in Figure 6a,b, it can be found that the PI interface temperature near the CNTs is much higher than that without CNTs, which can also be confirmed from Figure 6c,d. The lattice temperatures of the interface with CNTs integrated can be increased by 1500 K, 2200 K, and 2800 K with the three sub-pulses, whereas the lattice temperatures without CNTs integrated can only be increased by 450 K, 680 K, and 800 K, respectively. It can be seen that the CNTs can effectively increase the interface temperature when the same picosecond laser is used to irradiate the PI interface. Furthermore, Jin et al.’s research indicates that CNTs exhibit an extremely high light absorption rate (more than 95%) across a broad spectral range from ultraviolet to near-infrared, with little variation with wavelength [37]. The above results collectively indicate that CNTs can enhance the light absorption at the interface and effectively convert light energy into thermal energy, thereby causing the interface temperature of PI/CNTs to rise rapidly. The excellent light-to-heat conversion characteristic enables the utilization of lower-power lasers to complete the laser lift-off process, which can not only reduce the equipment cost but also significantly reduce the risk of thermal damage to the sensitive chip devices being lifted off, effectively improving process tolerance and product yield.

## 4. Conclusions

In this paper, the two-temperature model is applied to investigate the effects of three different ultra-fast lasers with different pulse intervals and CNTs on the PI film LLO process. This paper first investigates the effects of nanosecond, picosecond, and femtosecond lasers with different pulse intervals on the lattice temperature of the PI/CNT/glass interface at their respective commonly used pulse widths. The simulation results show that the lattice temperature increment with the picosecond laser with a pulse interval of 30 ps is approximately 2800 K, which is much higher than the lattice temperature increase with the nanosecond and femtosecond lasers. Subsequently, the influences of the CNTs at the PI/glass interface on the lattice temperature with picosecond laser irradiation are also investigated with a pulse width of 20 ps and a pulse interval of 30 ps. It can also be found that the lattice temperature of the interface with CNTs integrated is increased by up to about 2800 K, which is around 2000 K higher than the lattice temperature increment of the PI interface without CNTs with the same picosecond laser irradiation. Our findings indicate that integrating CNTs at the PI/glass interface can further lower the laser energy threshold and significantly reduce the risk of thermal damage during the LLO process, which may provide a new technology for ultra-fast laser debonding with lower energy.

## Data Availability

All the data supporting the findings of this study are presented in this article. Further inquiries can be directed to the corresponding author.

## References

[1] Huang Y., Wu H., Xiao L., Duan Y., Zhu H., Bian J., Ye D., Yin Z. (2019). Assembly and applications of 3D conformal electronics on curvilinear surfaces. Mater. Horiz..

[2] Joo H., Lee Y., Kim J., Yoo J.S., Yoo S., Kim S., Arya A.K., Kim S., Choi S.H., Lu N. (2021). Soft implantable drug delivery device integrated wirelessly with wearable devices to treat fatal seizures. Sci. Adv..

[3] Hassan M., Abbas G., Li N., Afzal A., Haider Z., Ahmed S., Xu X., Pan C., Peng Z. (2021). Significance of Flexible Substrates for Wearable and Implantable Devices: Recent Advances and Perspectives. Adv. Mater. Technol..

[4] Yang Y., Li Z., Yang S., Li Y., Huang J. (2020). Multiscale simulation study of laser sintering of inkjet-printed silver nanoparticle inks. Int. J. Heat Mass Transf..

[5] Zhai Y., Mathew L., Rao R., Xu D., Banerjee S.K. (2012). High-performance flexible thin-film transistors exfoliated from bulk wafer. Nano Lett..

[6] Meitl A.M., Zhu Z., Kumar V., Lee K.J., Feng X., Huang Y.Y., Adesida I., Nuzzo R.G., Rogers J.A. (2006). Transfer printing by kinetic control of adhesion to an elastomeric stamp. Nat. Mater..

[7] Hwang G.T., Im D., Lee S.E., Lee J., Koo M., Park S.Y., Kim S., Yang K., Kim S.J., Lee K. (2013). In vivo silicon-based flexible radio frequency integrated circuits monolithically encapsulated with biocompatible liquid crystal polymers. ACS Nano.

[8] Dagdeviren C., Su Y., Joe P., Yona R., Liu Y., Kim Y.S., Huang Y.A., Damadoran A.R., Xia J., Martin L.W. (2014). Conformable amplified lead zirconate titanate sensors with enhanced piezoelectric response for cutaneous pressure monitoring. Nat. Commun..

[9] Hallum G.E., Kürschner D., Eulenkamp C., Auer R., Hartmann B., Schulz W., Huber H.P. (2023). Indium tin oxide ultra-fast laser lift off ablation mechanisms and damage minimization. Opt. Express.

[10] Tian J., Huang J., Wang M., Yu K., Guo H., Luo W., Dong Y., Wang Y., Zhang Z., Zhang W. (2024). Laser Liftoff Enabled Batch Fabrication of Ultrathin Graphene Hall Devices. ACS Appl. Mater. Interfaces.

[11] Lv B., Ye L., Zhu X., Liu Y., Chuai S., Wang Z. (2024). Ultrasonic-assisted stripping of single-crystal SiC after laser modification. Ceram. Int..

[12] Joe D.J., Kim S., Park J.H., Park D.Y., Lee H.E., Im T.H., Choi I., Ruoff R.S., Lee K.J. (2017). Laser-material interactions for flexible applications. Adv. Mater..

[13] Park J., Sin Y., Kim J., Kim J. (2016). Dependence of adhesion strength between GaN LEDs and sapphire substrate on power density of UV laser irradiation. Appl. Surf. Sci..

[14] Zhu C., Guo D., Ye D., Jiang S., Huang Y.A. (2020). Flexible PZT-Integrated, Bilateral Sensors via Transfer-Free Laser Lift-Off for Multimodal Measurements. ACS Appl. Mater. Interfaces.

[15] Do Y.H., Jung W.S., Kang M.G., Kang C.Y., Yoon S.J. (2013). Preparation on transparent flexible piezoelectric energy harvester based on PZT films by laser lift-off process. Sens. Actuators Phys..

[16] Park K.I., Son J.H., Hwang G.T., Jeong C.K., Ryu J., Koo M., Choi I., Lee S.H., Byun M., Wang Z.L. (2014). Highly-efficient, flexible piezoelectric PZT thin film nanogenerator on plastic substrates. Adv. Mater..

[17] Lee H.E., Kim S., Ko J., Yeom H.I., Byun C.W., Lee S.H., Joe D.J., Im T.H., Park S.H., Lee K.J. (2016). Skin-like oxide thin-film transistors for transparent displays. Adv. Funct. Mater..

[18] Kim J.Y., Lee E.K., Jung J., Lee D.W., Yun Y., Chung J.W., Park J.I., Kim J.J. (2019). Densely cross-linked polysiloxane dielectric for organic thin-film transistors with enhanced electrical stability. J. Mater. Chem. C.

[19] Delmdahl B.R., Fricke M., Fechner B. (2014). Laser lift off systems for flexible-display production. J. Inf. Disp..

[20] Bian J., Zhou L., Yang B., Yin Z., Huang Y. (2020). Theoretical and experimental studies of laser lift off of nonwrinkled ultrathin polyimide film for flexible electronics. Appl. Surf. Sci..

[21] Luo H., Wang S., Wang C., Linghu C., Song J. (2021). Thermal Controlled Tunable Adhesive for Deterministic Assembly by Transfer Printing. Adv. Funct. Mater..

[22] Ke X., Zhang S., Chai Z., Jiang J., Xu Y., Tao B., Ding H., Wu Z. (2020). Flexible discretely-magnetized configurable soft robots via laser-tuned selective transfer printing of anisotropic ferromagnetic cells. Mater. Today Phys..

[23] Wang C., Linghu C., Nie S., Li C., Lei Q., Tao X., Zeng Y., Du Y., Zhang S., Yu K. (2020). Programmable and scalable transfer printing with high reliability and efficiency for flexible inorganic electronics. Sci. Adv..

[24] Bian J., Chen F., Ling H., Sun N., Hu J., Huang Y. (2022). Experimental and modeling study of controllable laser lift off via low-fluence multiscanning of polyimide-substrate interface. Int. J. Heat Mass Transf..

[25] Bian J., Chen F., Yang B., Hu J., Sun N., Ye D., Duan Y., Yin Z., Huang Y.A. (2020). Laser-Induced Interfacial Spallation for Controllable and Versatile Delamination of Flexible Electronics. ACS Appl. Mater. Interfaces.

[26] Pan H., Hu Y., Liu H., Guo Y., Kang Y., Wang W., Liu Y. (2024). Thermal Damage Mechanism and Micro-mechanical Property Analysis of Carbonate Rocks Under Laser Irradiation. Rock Mech. Rock Eng..

[27] Voronenkov V., Bochkareva N., Gorbunov R., Zubrilov A., Kogotkov V., Latyshev P., Lelikov Y., Leonidov A., Shreter Y. (2019). Laser slicing: A thin film lift off method for GaN-on-GaN technology. Results Phys..

[28] Avdizhiyan A., Janus W., Szpytma M., Slezak T., Przybylski M., Chrobak M., Roddatis V., Stupakiewicz A., Razdolski I. (2023). Ultra-fast Laser-Induced Dynamics of Non-Equilibrium Electron Spill-Out in Nanoplasmonic Bilayers. Nano Lett..

[29] Li J., Yan J., Jiang L., Yu J., Guo H., Qu L. (2023). Nanoscale multi-beam lithography of photonic crystals with ultra-fast laser. Light Sci. Appl..

[30] Feng J., Wang J., Liu H., Sun Y., Fu X., Ji S., Liao Y., Tian Y. (2024). A Review of an Investigation of the Ultrafast Laser Processing of Brittle and Hard Materials. Materials.

[31] Kim Y., Noh Y., Park S., Kim B.-K., Kim H.J. (2020). Ablation of polyimide thin-film on carrier glass using 355 nm and 37 ns laser pulses. Int. J. Heat Mass Transf..

[32] Leahu G., Li Voti R., Larciprete M.C., Sibilia C., Bertolotti M., Nefedov I., Anoshkin I.V. (2015). Thermal Characterization of Carbon Nanotubes by Photothermal Techniques. Int. J. Thermophys..

[33] Bertolotti M., Ferrari A., Liakhou G.L., Li Voti R., Marras A., Ezquerra T.A., Balta-Calleja F.J. (1995). Thermal anisotropy of polymer carbon fiber composites as revealed by photodeflection methods. J. Appl. Phys..

[34] He K., Ren Y., Dai Z., Zhang J., Tu X., Cheng L., Xin Z., Cai L., Ye Y. (2023). Theoretical and experimental study of ablation of fused silica by femtosecond laser bursts. Opt. Commun..

[35] Long H., Feng X., Wei Y., Yu T., Fan S., Ying L., Zhang B. (2017). Carbon nanotube assisted Lift off of GaN layers on sapphire. Appl. Surf. Sci..

[36] Zhang Q., Chen G., Yoon S.F., Ahn J., Wang S.G., Zhou Q., Wang Q., Li J.Q. (2002). Thermal conductivity of multiwalled carbon nanotubes. Phys. Rev. B.

[37] Jin Y., Zhang T., Zhao J., Zhao Y., Liu C., Song J., Hao X., Wang J., Jiang K., Fan S. (2021). Spray coating of a perfect absorber based on carbon nanotube multiscale composites. Carbon.

